# Synthetic approach of ternary magnesium niobate (Mg–Nb–O) compounds

**DOI:** 10.1038/s41598-021-95690-4

**Published:** 2021-08-09

**Authors:** Md. Wasikur Rahman

**Affiliations:** Department of Chemical Engineering, Jashore University of Science and Technology, Jashore, 7408 Bangladesh

**Keywords:** Chemistry, Energy science and technology, Materials science, Nanoscience and technology

## Abstract

Magnesium based niobium oxides (Mg–Nb–O) were prepared by solid-state reactions owing to understand the function of transition metal oxides as promoters/catalysts for practical application. Magnesium niobate (Mg_3_Nb_6_O_11_) was synthesized for the first time in nearly pure form reported in this context. MgNb_2_O_6_ and Mg_4_Nb_2_O_9_ were prepared in oxidizing conditions; on the contrary, Mg_3_Nb_6_O_11_ preferred reducing environment. Stoichiometric mixtures of the precursor materials MgO, Nb_2_O_5_ and/or metallic Nb were annealed for the syntheses which revealed the effect of temperature on phase formation, reaction kinetics and heat of reaction. The products were examined by ex-situ, in-situ X-ray diffraction (XRD) and differential scanning calorimetry (DSC). Crystallographic parameters of various binary and ternary compounds (Mg/Nb/O) formed in different calcination conditions, were extracted by Rietveld method. In-situ experiment results in single step reaction for the MgNb_2_O_6_ synthesis and the heat of formation of the solid-state reaction obtained to be minimum (93 kJ/mol). In contrast, the formation of Mg_4_Nb_2_O_9_ and Mg_3_Nb_6_O_11_ compounds towards pure phases rather complicated due to multistep reactions and corresponding heat of formation were estimated to be 140 and 190 kJ/mol. Experimental results have been discussed based on kinetic and thermodynamic constrains.

## Introduction

Hydrogen storage represents an important step in the development of hydrogen economy and various storage systems were reported in the literatures. Among these systems, magnesium hydride (MgH_2_) is an interesting material for H_2_ storage^1,2^ owing to high abundance in the lithosphere, cost-effective and less toxic properties^3,4^. However, higher temperature is required in H_2_ absorption/desorption cycles which are characterized by relatively slow reaction kinetics.

Different strategies have been proposed to overcome these problems such as ball milling and addition of catalysts, particularly, transition metal oxides (TMO), metals, alloys, etc.^5^. Huge interest in TMO was reported in the literatures. It has been revealed from the reviews that niobia (Nb_2_O_5_) is a competent additive even though its activity is still an unresolved issue as a promoter/additive to alter the reaction kinetics of the system^6–9^.

Milling of MgH_2_ with Nb_2_O_5_ influenced H_2_ absorption/desorption kinetics and Mg–Nb–O ternary compounds were formed during H_2_ sorption cycles^10–12^. In particular, a reactive pathway model was proposed by Friedrichs et al.^13^ point out the fact that the reduction of Nb_2_O_5_ into metallic Nb, followed by successive formation of magnesium based oxides (Mg–Nb–O) which facilitate H_2_ transport into the solid structures. Recently, the effect of the presence of Mg–Nb mixed oxide compounds (MgNb_2_O_6_, Mg_4_Nb_2_O_9_ and Mg_3_Nb_6_O_11_) on H_2_ absorption properties of MgH_2_ was investigated^9,14,15^.

A number of possibilities for the formation of binary and ternary compounds with various oxidation states of Nb are available in the literatures^16–25^. Abbattista et al.^26^ obtained an orthorhombic phase of MgNb_2_O_3.6_ from reduction of MgNb_2_O_6_. Marinder et al.^27^ also reported the preparation of Mg_3_Nb_6_O_11_ from precursor mixtures of MgO/NbO_2_ with different molar ratios of Mg:Nb. The synthesis of some ternary compounds, e.g., MgNb_2_O_6_, Mg_4_Nb_2_O_9_, Mg_3_Nb_6_O_11_ and Mg_5_Nb_4_O_15_ were reported elsewhere^24,28^. Pagola et al.^24^ proposed an effective method of solid-state synthetic reactions Mg–Nb oxides basically annealing commercial precursor materials. During preparation of MgNb_2_O_6_ compound from the starting materials (MgO and Nb_2_O_5_), MgNb_2_O_6_ phase of columbite structure is usually obtained including corundum-like Mg_4_Nb_2_O_9_^29^. Crystallographic, microstructural and morphological features of MgNb_2_O_6_ and Mg_4_Nb_2_O_9_ compounds were reported elsewhere^30^. Moreover, they are stable phases at room temperature explored by You et al.^31^. A crystallographic investigation of the orthorhombic columbite-like MgNb_2_O_6_ phase was done and the fine-structural features of this compound were established from neutron diffraction investigation^24^. Mg_4_Nb_2_O_9_ shows corundum-type (α-Al_2_O_3_) structure resulting from order of Mg^2+^ and Nb^5+^ ions^32,33^. Nonetheless, proper selection of starting materials, calcination conditions to be optimized and reaction mechanism aiming to have pure phases not yet studied in details.

The present work aims to explore in details the synthetic routes for the preparation of three Mg–Nb–O compounds (e.g., MgNb_2_O_6_, Mg_4_Nb_2_O_9_ and Mg_3_Nb_6_O_11_) in nearly pure phases and the products were characterized by ex-situ and in-situ XRD and DSC experiments.

## Methodology

### Materials and methods

Commercially available MgO, Nb_2_O_5_ and Nb (Sigma-Aldrich, Germany) were employed as precursor materials for the solid-state synthesis. MgNb_2_O_6_ and Mg_4_Nb_2_O_9_ were prepared by annealing MgO/Nb_2_O_5_ with stoichiometric ratio in oxidizing atmosphere. A mixture of MgO/Nb_2_O_5_/Nb powders was heated in evacuated quartz ampoules for Mg_3_Nb_6_O_11_ preparation. The mixtures were annealed from room temperature (RT) to 473, 673, 873, 1073, 1273 and 1473 K for 24 h and heating rate was set at 10 K/min for all the cases.

### Characterization

#### XRD analysis

Structural analysis of the as-prepared materials was carried out by ex-situ XRD diffractometer (Panalytical) with a radiation source of Cu Kα. A reaction chamber (Anton Paar XRK 900) was employed for in-situ XRD study. XRD patterns were recorded in isothermal conditions following suitable temperature step programs with a step size of 0.017° for 6 min from RT to 1173 K at 10 K/min. The experiment was carried out in a steel-made sample holder under vacuum condition and its thermal expansion was estimated to be shifted nearly 0.1° with a reference of α-quartz. In fact, the shifting of the observed peaks did not alter the lattice constants considerably. Therefore, during in-situ XRD, the surface of the powdered sample moved significantly from the goniometer centre. Continuous vacuum was introduced into the reaction chamber. The diffraction patterns were documented as a function of time and the experiments of the preparation of MgNb_2_O_6_, Mg_4_Nb_2_O_9_ and Mg_3_Nb_6_O_11_ required 10–18 h.

#### DSC analysis

Differential scanning calorimetry (DSC) thermograms were recorded due to the synthesis of Mg–Nb oxides from RT to 1473 K at 10 K/min using a calorimeter (Setaram) of high temperature with a flow of He and Ar. The reference (αAl_2_O_3_, ca. 0.2 g) and mixtures of precursor materials were loaded in Pt crucibles.

Crystallographic information of the powder samples built on XRD patterns were evaluated by using MAUD (Material Analysis Using Diffraction). MAUD is oriented to the studies of material science. It is a general analytical program based on diffraction/reflectivity data and mainly supports Rietveld method^34^.

## Results and discussion

### Ex-situ experiment

**Figure 1:**
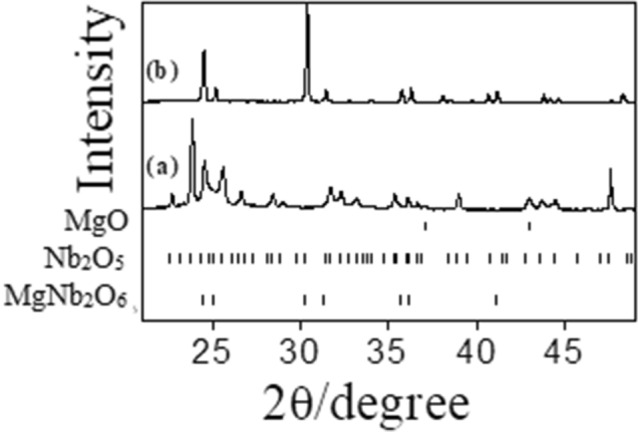
Ex-situ XRD patterns obtained during MgNb_2_O_6_ preparation: (**a**) 298 K and (**b**) 1473 K.

**Figure 2:**
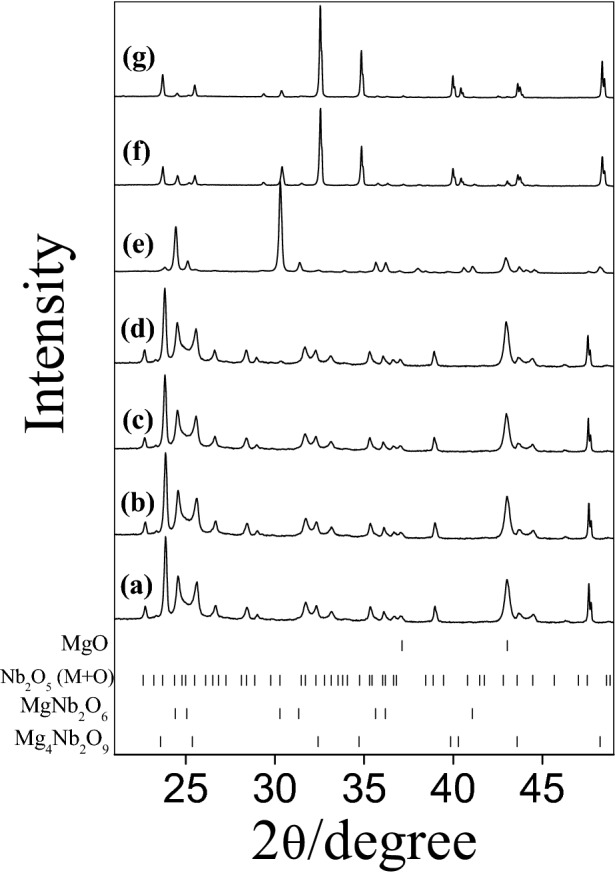
Ex-situ XRD patterns obtained during Mg_4_Nb_2_O_9_ preparation at (**a**) 298 K, (**b**) 473 K, (**c**) 673 K, (**d**) 873 K, (**e**) 1073 K, (**f**) 1273 K and (**g**) 1473 K.

**Figure 3:**
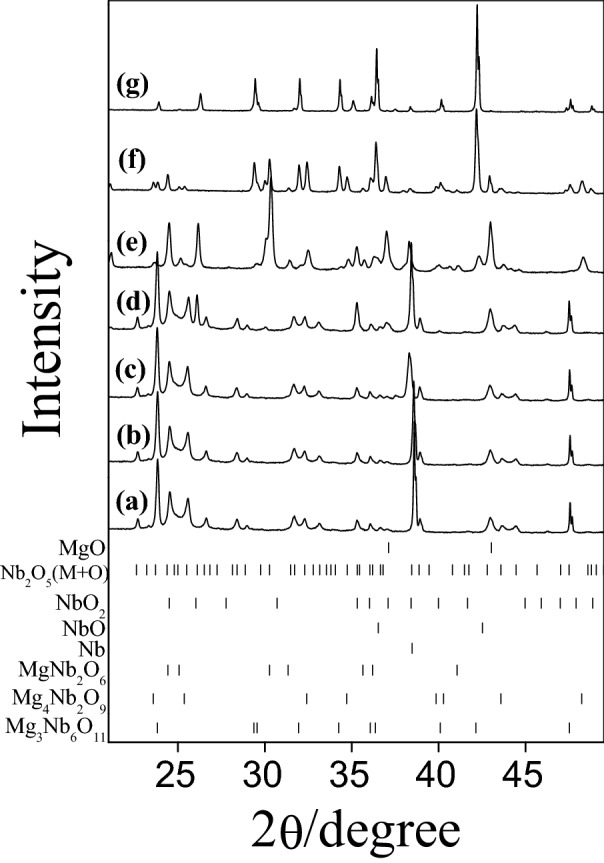
Ex-situ XRD patterns obtained during Mg_3_Nb_6_O_11_ preparation at (**a**) 298 K, (**b**) 473 K, (**c**) 673 K, (**d**) 873 K, (**e**) 1073 K, (**f**) 1273 K and (**g**) 1473 K.

Solid-state synthetic phase evolution is a function of temperature during heating the precursor materials. XRD patterns corresponding to ex-situ measurements of the as-prepared samples were recorded at RT. The XRD patterns monitored along the process of formation of MgNb_2_O_6_, Mg_4_Nb_2_O_9_ and Mg_3_Nb_6_O_11_ are reported in the Figs. 1, 2 and 3, respectively and the phase abundance estimated by Rietveld refinement has been inserted in Tables 1, 2 and 3. XRD patterns of the samples were recorded from 10° to 90°; however, due to simplicity and more clear presentation, only the data from 20° to 50° are reported. A detailed description of phase composition of various binary and ternary oxides grown up with increasing temperatures towards solid state synthesis of pure Mg–Nb–O compounds was evaluated by Rietveld refinement with excellent fittings (R% = 1–4%).

**Table 1:** Abundance of MgNb_2_O_6_ compound obtained by Rietveld refinement of XRD patterns at 298 and 1473 K (MC = monoclinic, OR = Orthorombic, O_6_ = MgNb_2_O_6_).

T/K	Phase composition (wt%)				R%
	Nb_2_O_5_ MC	Nb_2_O_5_ OR	MgO	O_6_	
298	80	7	13	0	2.45
1473	3	0	0	97	1.86

**Table 2:** Abundance of Mg_4_Nb_2_O_9_ compound obtained by Rietveld refinement of XRD patterns at different temperatures (MC = monoclinic, OR = Orthorombic, O_6_ = MgNb_2_O_6_, O_9_ = Mg_4_Nb_2_O_9_).

T/K	Phase composition (wt%)					R%
	Nb_2_O_5_ MC	Nb_2_O_5_ OR	MgO	O_6_	O_9_	
298	61	4	35	0	0	2.58
473	62	4	34	0	0	2.55
673	62	4	34	0	0	2.43
873	58	5	30	7	0	2.88
1073	9	0	25	64	2	1.75
1273	2	0	2	3	93	1.31
1473	0	0	0	5	95	2.16

**Table 3:** Abundance of Mg_3_Nb_6_O_11_ compounds obtained by Rietveld refinement of XRD patterns at different temperatures (MC = monoclinic, OR = Orthorombic, O_6_ = MgNb_2_O_6_, O_9_ = Mg_4_Nb_2_O_9_, O_11_ = Mg_3_Nb_6_O_11_).

T/K	Phase composition (wt%)									R%
	Nb_2_O_5_ MC	Nb_2_O_5_ OR	MgO	Nb	NbO	NbO_2_	O_6_	O_9_	O_11_	
298	64	5	15	16	0	0	0	0	0	3.58
473	70	4	15	11	0	0	0	0	0	3.60
673	72	5	14	9	0	0	0	0	0	3.28
873	64	4	15	8	2	7	0	0	0	4.07
1073	0	0	8	1	16	11	39	15	10	0.58
1273	0	0	2	0	6	0	11	29	52	0.26
1473	0	0	0	0	0	0	8	0	92	1.76

During the synthesis of MgNb_2_O_6_ (Fig. 1 and Table 1), the binary precursor materials (MgO and Nb_2_O_5_) reacted according to the following reaction:

1 $${\text{MgO}}\, + \,{\text{Nb}}_{2} {\text{O}}_{5} \to {\text{MgNb}}_{2} {\text{O}}_{6}$$

Detailed synthetic method of MgNb_2_O_6_ was discussed in the previous study^35^. In the current issue XRD patterns at RT and maximum reaction temperature 1473 K are shown (Fig. 1a,b). In fact, no solid state reactions were carried out from RT to less than 673 K followed by calcination at 673–1073 K; the evolution of columbite-like MgNb_2_O_6_ phase was observed. A nearly pure MgNb_2_O_6_ phase was obtained at 1273 K and the solid-state reaction of the desired compound was completed at 1473 K (97 wt%) excepting traces of unreacted more stable monoclinic Nb_2_O_5_ (Table 1).

In the case of Mg_4_Nb_2_O_9_ preparation (Fig. 2 and Table 2) the precursor oxides are basically unaffected by the treatment in the range RT-873 K (Fig. 2a,b,c,d). The MgNb_2_O_6_ phase appeared as intermediate after annealed at 1073 K (Fig. 2e). With increasing temperature up to 1273 K, diffraction patterns showed simultaneous presence of various binary and ternary compounds (Mg/Nb/O) (Fig. 2f). In this step, the fraction of MgNb_2_O_6_ decreased and a considerable amount of the corundum-type Mg_4_Nb_2_O_9_ was formed. At higher test temperature (1473 K), almost pure Mg_4_Nb_2_O_9_ phase was observed (Fig. 2g). The whole process can be described by the following stoichiometry:

2 $$4{\text{MgO}}\, + \,{\text{Nb}}_{2} {\text{O}}_{5} \to {\text{Mg}}_{4} {\text{Nb}}_{2} {\text{O}}_{9}$$

However, the final formation of the mixed phase which started at 1473 K occurs on the basis of a reaction between MgO and MgNb_2_O_6_ according to the following reaction:

3 $$3{\text{MgO}}\, + \,{\text{MgNb}}_{2} {\text{O}}_{6} \to {\text{Mg}}_{4} {\text{Nb}}_{2} {\text{O}}_{9}$$

The phase evolution leading to Mg_3_Nb_6_O_11_ is more complicated than in the previous two cases owing to the presence of more reactive metallic Nb in the precursor mixture. The chemical composition remains nearly constant up to 673 K (Fig. 3a,b). Upon annealing at 873 K, niobium oxides (NbO_2_ and NbO) of lower oxidation states were appeared.

The formation of niobium monoxide (NbO) and dioxide (NbO_2_) can be explained considering that the synthesis was carried out in static vacuum in order to avoid oxidation of metallic Nb. However, in these conditions, for a semiconducting oxide like Nb_2_O_5_, oxygen depletion easily occurs and metallic Nb is thus partially oxidized by the released oxygen (Reactions 4 and 5). Similar effects were also reported for Mg–Ng–O compounds^16^.

4 $${\text{Nb}}_{2} {\text{O}}_{5} \to {\text{Nb}}_{2} {\text{O}}_{{5{-}x}} + {\text{x}}/2{\text{O}}_{2}$$

5 $${\text{xO}}_{2} + {\text{Nb}} \to 2{\text{NbO}}_{{\text{x}}}$$

Only at higher temperature (1073 K) MgNb_2_O_6_ appeared as a predominant phase (Fig. 2e). In addition, at this temperature various peaks related to Mg_3_Nb_6_O_11_ were observed and its phase quantity gradually increased with the temperature. With raising the synthetic temperature to 1273 K, the abundance of both MgNb_2_O_6_ and binary oxides decreased and some amount of Mg_4_Nb_2_O_9_ was observed together with the desired Mg_3_Nb_6_O_11_ phase (Fig. 3f). Then at higher temperature (1473 K), approximately a single phase of the ternary compound (92 wt%) was obtained with some contamination only by MgNb_2_O_6_ (8 wt%) (Fig. 3g).

### In-situ experiment

**Figure 4:**
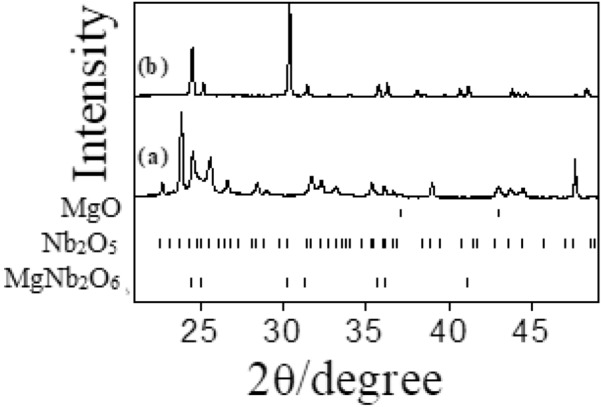
In-situ XRD patterns obtained during MgNb_2_O_6_ preparation at (**a**) 298 K and (**b**) 1173 K.

**Figure 5:**
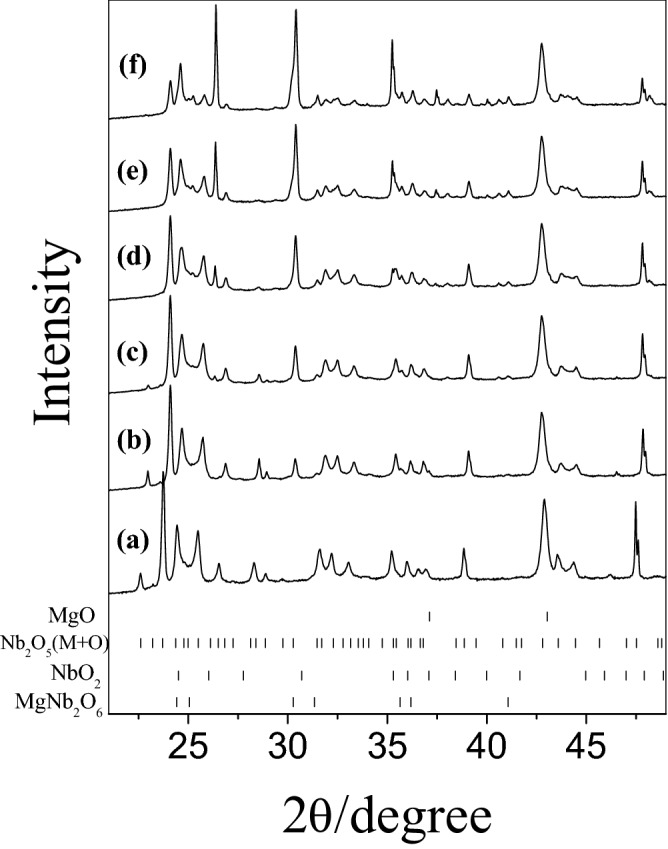
In-situ XRD patterns obtained during Mg_4_Nb_2_O_9_ preparation at (**a**) 298 K, (**b**) 1073 K, (**c**) 1098 K, (**d**) 1123 K, (**e**) 1148 K and (**f**) 1173 K.

**Figure 6:**
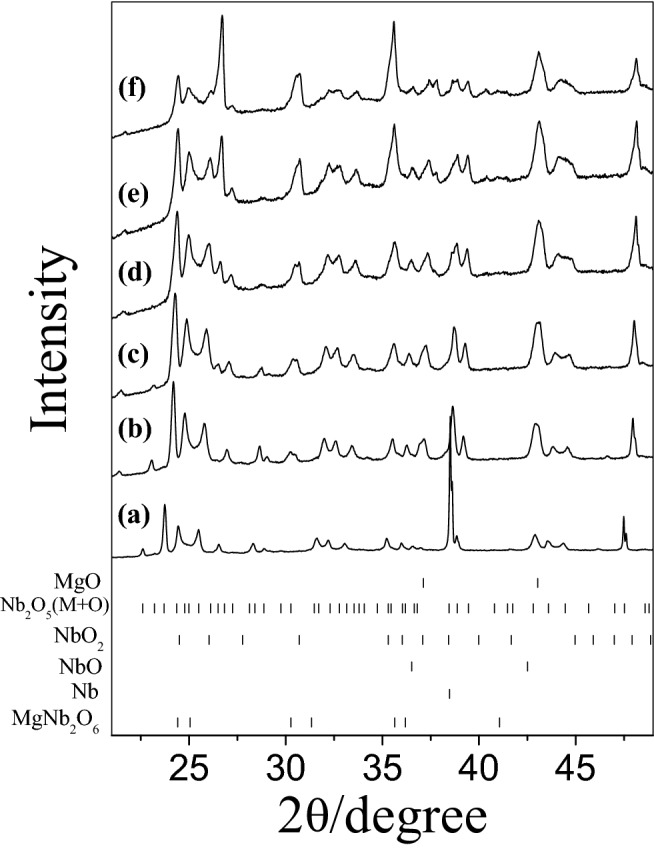
In-situ XRD patterns obtained during Mg_3_Nb_6_O_11_ preparation at (**a**) 298 K, (**b**) 1073 K, (**c**) 1098 K, (**d**) 1123 K, (**e**) 1148 K and (**f**) 1173 K.

In-situ experiments, aimed to study kinetics of the preparation of Mg–Nb–O compounds, were carried out from RT to 1173 K in isothermal conditions (Figs. 4, 5, 6) and corresponding amount of phases obtained by Rietveld analysis are reported in Tables 4, 5 and 6.

**Table 4:** Abundance of MgNb_2_O_6_ phase compound obtained by Rietveld refinement of in–situ XRD patterns (MC = monoclinic, OR = Orthorombic, O_6_ = MgNb_2_O_6_).

T/K	Phase composition (wt%)						R%
	Nb_2_O_5_ MC	Nb_2_O_5_ OR	NbO	NbO_2_	MgO	MgNb_2_O_6_	
298	78	9	0	0	13	0	1.60
1173	55	0	0	17	8	20	1.33

**Table 5:** Abundance of Mg_4_Nb_2_O_9_ compound obtained by Rietveld refinement of in–situ XRD patterns (MC = monoclinic, OR = Orthorombic, O_6_ = MgNb_2_O_6_).

T/K	Phase composition (wt%)					R%
	Nb_2_O_5_ MC	Nb_2_O_5_ OR	MgO	NbO_2_	MgNb_2_O_6_	
298	55	7	38	0	0	1.57
1073	54	6	37	0	3	1.56
1098	53	3	38	1	5	1.43
1123	51	2	32	3	12	2.03
1148	54	0	27	4	15	2.11
1173	45	0	24	5	26	2.30

**Table 6:** Abundance of Mg_3_Nb_6_O_11_ compound obtained by Rietveld refinement of in–situ XRD patterns (MC = monoclinic, OR = Orthorombic, O_6_ = MgNb_2_O_6_).

T/K	Phase composition (wt%)							R%
	Nb_2_O_5_MC	Nb_2_O_5_OR	Nb	NbO	NbO_2_	MgO	MgNb_2_O_6_	
298	62	7	15	0	0	16	0	2.70
1073	64	6	2	4	7	15	2	2.10
1098	65	5	0	4	9	14	3	1.85
1123	62	2	0	5	11	15	5	1.57
1148	52	0	0	4	16	14	14	2.34
1173	47	0	0	3	20	12	18	3.92

Considering that no appreciable reactions take place among the parent materials at lower temperature during in-situ experiments. After an initial pattern acquisition at RT, the solid-state synthetic temperature was increased directly to 973 K during MgNb_2_O_6_ preparation and up to 1073 K in the other two cases (Figs. 4, 5, 6 and Tables 4, 5, 6).

In-situ XRD data for MgNb_2_O_6_ preparation are shown in Fig. 4 and corresponding phase compositions are inserted in Table 4. In this case the MgNb_2_O_6_ phase appeared at 1123 K following Reaction 1 and the abundance of this phase increased with the temperature as proved by ex-situ measurements. Phase evolution with temperature (298–1173 K) are described in our previous work^35^.

In the case of in-situ Mg_4_Nb_2_O_9_ preparation (Fig. 5) and resultant phase compositions are introduced in Table 5, where only the MgNb_2_O_6_ phase was detected for the reasons reported earlier. The presence of this phase, however, confirms the compound representing a key step in the Mg_4_Nb_2_O_9_ phase formation (Reactions 1 and 3).

Similarly, MgNb_2_O_6_ phase was observed as an early stage during in-situ study of Mg_3_Nb_6_O_11_ (Fig. 6) and respective phase contents are placed in Table 6. In fact, this phase evolution is much more complicated than the other two phases as also detected in ex-situ experiment, might be owing to the addition of more reactive metallic Nb. The evolution of the various phases indicates that, even in vacuum condition, metallic Nb is easily oxidized at lower temperatures hindering the formation of Mg_3_Nb_6_O_11_.

Through this approach of the ternary compound synthesis, in contrast to ex-situ method, no pure phases were appeared due to temperature and kinetic restrictions. In fact, the highest temperature that can be reached by the in-situ equipment is lower than that of ex-situ preparation because of instrumental limitation. Moreover, incomplete phase transformation is determined by the reaction time inferior to the ex-situ experiments; however, the in-situ test is very important to comprehend the kinetics of the solid-state synthesis. Besides, low-valence niobium oxides (NbO and NbO_2_) were evidenced for all the cases during in-situ experiment focus the fact of vacuum conditions at which the reactions were carried out.

**Figure 7:**
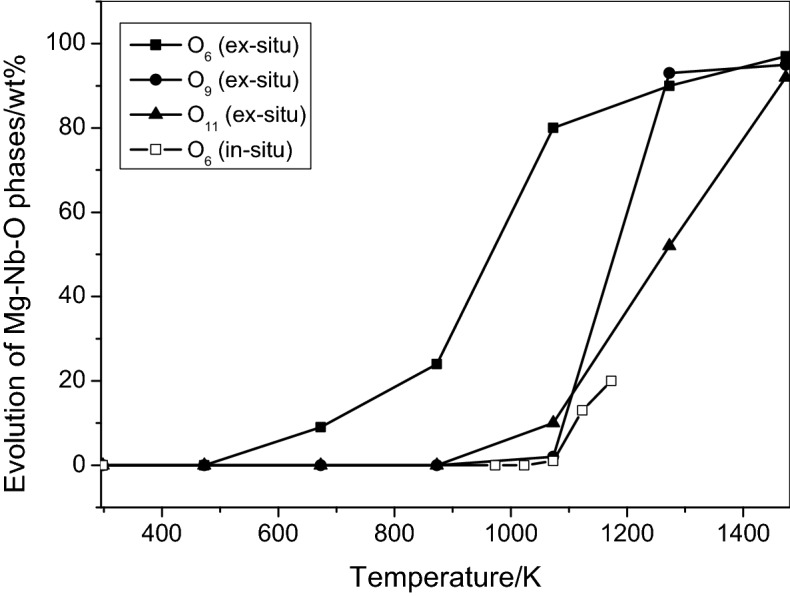
Comparison of the evolution of various ternary Mg-Nb–O phases (O_6_ = MgNb_2_O_6_, O_9_ = Mg_4_Nb_2_O_9_ and O_11_ = Mg_3_Nb_6_O_11_) with temperature during ex-situ and in-situ experiments.

The evolution of the various Mg–Nb oxide phases during ex-situ experiments are discussed earlier and compared them to the in-situ outcomes. Since it was not possible to obtain the Mg_4_Nb_2_O_9_ and Mg_3_Nb_6_O_11_ phases with the in-situ approach; a comparison between the two approaches can be done only for MgNb_2_O_6_. Nevertheless, the following considerations can be done:

Nearly 24 wt% of the MgNb_2_O_6_ phase was obtained at 873 K and 1073–1173 K by ex-situ and in-situ tests, respectively point out that in-situ measurement required higher temperature (Fig. 7, Tables 1 and 4)^35^. This indicates that MgNb_2_O_6_ formation is promoted by an oxidizing ambient. Moreover, the reasons behind Mg_4_Nb_2_O_9_ that was not appeared during in-situ experiments can be explained as the growing of the compound started at higher temperature. This evidence also can be interpreted that the formation of Mg_4_Nb_2_O_9_ is composed, at least, by two steps. Both the experiments indicate that the MgNb_2_O_6_ phase represents the first step during Mg_4_Nb_2_O_9_ preparation (Reactions 2 and 3). This phase then evolved to the final product at higher temperature. On the contrary, Mg_3_Nb_6_O_11_ formation is not parallel to the others and its mechanism is still an open question. Ex-situ experiment shows that the Mg_4_Nb_2_O_9_ and Mg_3_Nb_6_O_11_ phases were simultaneously appeared at about 1073 K and at this temperature, Mg_4_Nb_2_O_9_ formation is favoured (15%) rather than Mg_3_Nb_6_O_11_ (10%). In-situ measurement clearly shows that the grown-up of MgNb_2_O_6_ and Mg_4_Nb_2_O_9_ compounds were hampered in vacuum conditions. Moreover, they possibly react as follows (Reaction 6) at higher temperature to form Mg_3_Nb_6_O_11_ as evidenced by the quantity of the reactants obtained from Rietveld analysis decreased and that of the product increased (1273–1473, Table 3):

6 $$2{\text{MgNb}}_{2} {\text{O}}_{6} + {\text{Mg}}_{4} {\text{Nb}}_{2} {\text{O}}_{9} + {\text{NbO}}_{2} \to 2{\text{Mg}}_{3} {\text{Nb}}_{6} {\text{O}}_{11} + {\text{NbO}}$$

In addition, during the formation of Mg_3_Nb_6_O_11_ compound, the MgNb_2_O_6_ and Mg_4_Nb_2_O_9_ represent the intermediate steps which evolve to the final product at higher temperature according to the following reactions (Reaction 7 and 8):

7 $${\text{MgNb}}_{2} {\text{O}}_{6} + 2{\text{MgO}}\, + \,{\text{Nb}}_{2} {\text{O}}_{5} + 4{\text{Nb}} \to {\text{Mg}}_{3} {\text{Nb}}_{6} {\text{O}}_{11} + 2{\text{NbO}}$$

8 $${\text{Mg}}_{4} {\text{Nb}}_{2} {\text{O}}_{9} + 2{\text{MgO}} + 3{\text{Nb}}_{2} {\text{O}}_{5} + 8{\text{Nb}} \to 2{\text{Mg}}_{3} {\text{Nb}}_{6} {\text{O}}_{11} + 4{\text{NbO}}$$

Taking into account Reactions 4 and 5, the previous mechanism also explains why oxides with low oxidation states (NbO and NbO_2_) are always observed during the preparation of Mg_3_Nb_6_O_11_.

### DSC studies

**Figure 8:**
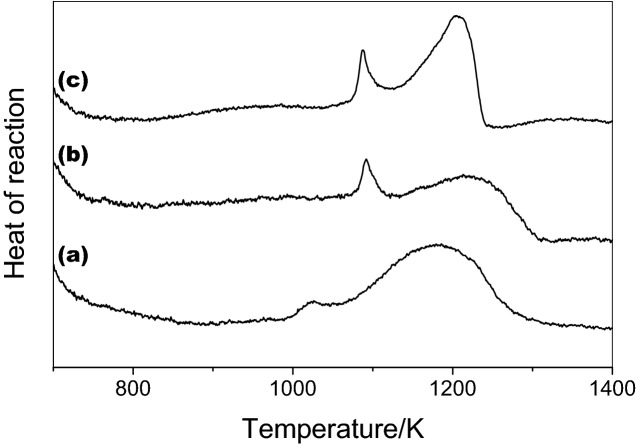
DSC curves for Mg–Nb–O ternary phases: (**a**) MgNb_2_O_6_, (**b**) Mg_4_Nb_2_O_9_ and (**c**) Mg_3_Nb_6_O_11_.

The preparation of the Mg–Nb–O compounds was studied by DSC analysis (Fig. 8) monitoring the final products by XRD with the aim of measuring the heat of reaction of the synthesis during the experiment. In the case of MgNb_2_O_6_ and Mg_4_Nb_2_O_9_, pure phases were observed at the highest test temperatures; whereas, a yield of 70% was obtained for Mg_3_Nb_6_O_11_ (Figure not attached due to simplicity).

Heating the starting materials for the synthesis up to 1400 K, three curves are obtained as shown in Fig. 8. All curves are characterized by exothermic signal spreads on a very large range of temperature (~ 200 K). Two peaks for each compound can be observed. In the case of MgNb_2_O_6_, the presence of two peaks nearly at 1090 and 1180 K (Fig. 8a) suggests that the overall process for the compound formation is complicated than the simple solid-state reaction reported earlier (Reaction 1). The presence of the two steps in the synthetic reaction can be interpreted through the following arguments:

The heating method connected to the DSC measurement is quite similar to that of the in-situ experiment where it was carried out in the reducing environment. In-situ results in fact also point out that the reaction is kinetically low and the intermediate products were formed as NbO and NbO_2_ in the range of 1023–1173 K.

The low temperature peaks in the DSC thermograms can be explained on the basis of the formation of such intermediates which, in this particular case, further reacted at higher temperature since they were not detected by XRD of the final product. A similar behaviour was revealed from the DSC curves for Mg_4_Nb_2_O_9_ and Mg_3_Nb_6_O_11_ (Fig. 8b,c). In these cases two peaks were also observed but they were shifted at higher temperature (1090 K for the both, and 1220 K and 1205 K for the Mg_4_Nb_2_O_9_ Mg_3_Nb_6_O_11_, respectively) (Fig. 8) indicating a multi-step pathway in the ternary oxide formation.

**Figure 9:**
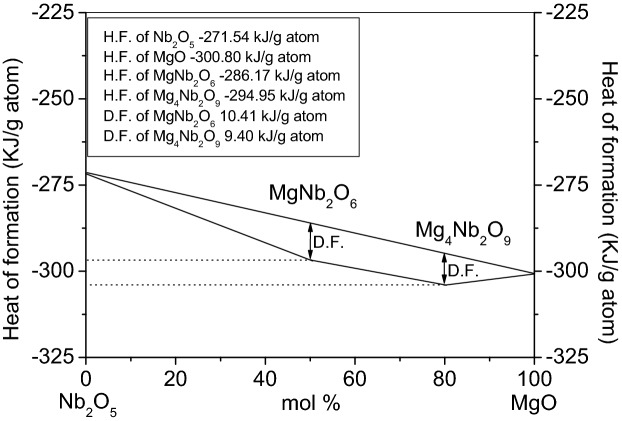
Heat of formation (H.F.) for MgNb_2_O_6_ and Mg_4_Nb_2_O_9_ (D.F. = Driving Force).

The heat of formation (HF), in the case of MgNb_2_O_6_, was obtained to be minimum (93 kJ/mol) calculated from the DSC results. In addition, the HF of Mg_4_Nb_2_O_9_ and Mg_3_Nb_6_O_11_ compounds towards pure phases were found 140 and 190 kJ/mol, respectively. Considering that the formation reaction is fully accomplished for MgNb_2_O_6_ and Mg_4_Nb_2_O_9_. Moreover, it is possible to obtain the HF for the two oxides from the DSC curves (Fig. 9) which results in − 286.17 kJ/g atom and − 294.95 kJ/g atom for MgNb_2_O_6_ and the Mg_4_Nb_2_O_9_, respectively. The driving force (DF) of the compounds can be calculated from available database when the synthesis is performed starting from MgO and Nb_2_O_5_ (Fig. 9).

This DF values are very close to the cases being 10.41 kJ/g atom and 9.40 kJ/g atom for the MgNb_2_O_6_ and Mg_4_Nb_2_O_9_, respectively thus MgNb_2_O_6_ representing the more stable phase in agreement with XRD results which clearly showed that during Mg_4_Nb_2_O_9_ preparation, the MgNb_2_O_6_ was always obtained as intermediate. Similar calculation for MgNb_2_O_6_ was reported earlier by our group^35^.

## Conclusions

In this work three Mg–Nb–O compounds of MgNb_2_O_6_, Mg_4_Nb_2_O_9_ and Mg_3_Nb_6_O_11_ were successfully prepared by solid-state reactions and the formation mechanism was characterized by means of XRD performed both in-situ and ex-situ. XRD analysis shows that the solid-state reactions leading to the ternary compounds are kinetically slow; therefore, high temperature with long time was needed for calcination of the precursor materials to obtain nearly pure phases. Moreover, the formation of MgNb_2_O_6_ and Mg_4_Nb_2_O_9_ was hampered when the synthesis was carried out in absence of oxygen. XRD also shows that MgNb_2_O_6_ represents an intermediate in the Mg_4_Nb_2_O_9_ and Mg_3_Nb_6_O_11_ formation. For the first time the heat of formation for the MgNb_2_O_6_ and Mg_4_Nb_2_O_9_ phases were estimated by means of DSC analysis which shows that the MgNb_2_O_6_ is thermodynamically more stable phase. The as-prepared three pure Mg–Nb–O phases, which are utilized as catalysts/additives to improve H_2_ sorption kinetics of MgH_2_ towards practical application, will be discussed in the forthcoming issues.

## Supplementary Information

Supplementary Information.
