# Supplementary material for: Synthetic approach of ternary magnesium niobate (Mg–Nb–O) compounds
Source: Sci Rep. 2021 Aug 9;11:16065. doi: 10.1038/s41598-021-95690-4 (PMC8352938; doi:10.1038/s41598-021-95690-4)
Supplement: Supplementary file 1 — Supplementary Information. [file 41598_2021_95690_MOESM1_ESM.pdf]

Supplemental Data

Tables 1 and 4 inserted in the manuscript are corresponded to Ex-situ and In-situ XRD results obtained from the solid state formation of MgNb2O6. The ternary compound is prepared from the precursors MgO and Nb2O5 in the temperature range 298-1473 K for Ex-situ and 298-1173 K for In-situ XRD. Total 13 XRD patterns were refined with Rietveld method and the data extracted from the refinement are inserted in the Tables. Only 2 fittings, for example, are presented in the following Figures (S1 for Ex-situ and S2 for In-situ). The others are available will be provided upon request.

$MgNb_2O_6$ specimen is almost totally single phase and shows the normal trend in the sq. root of counts plot due to a high level of background typical of multichannel analyzers (X'celeraton).

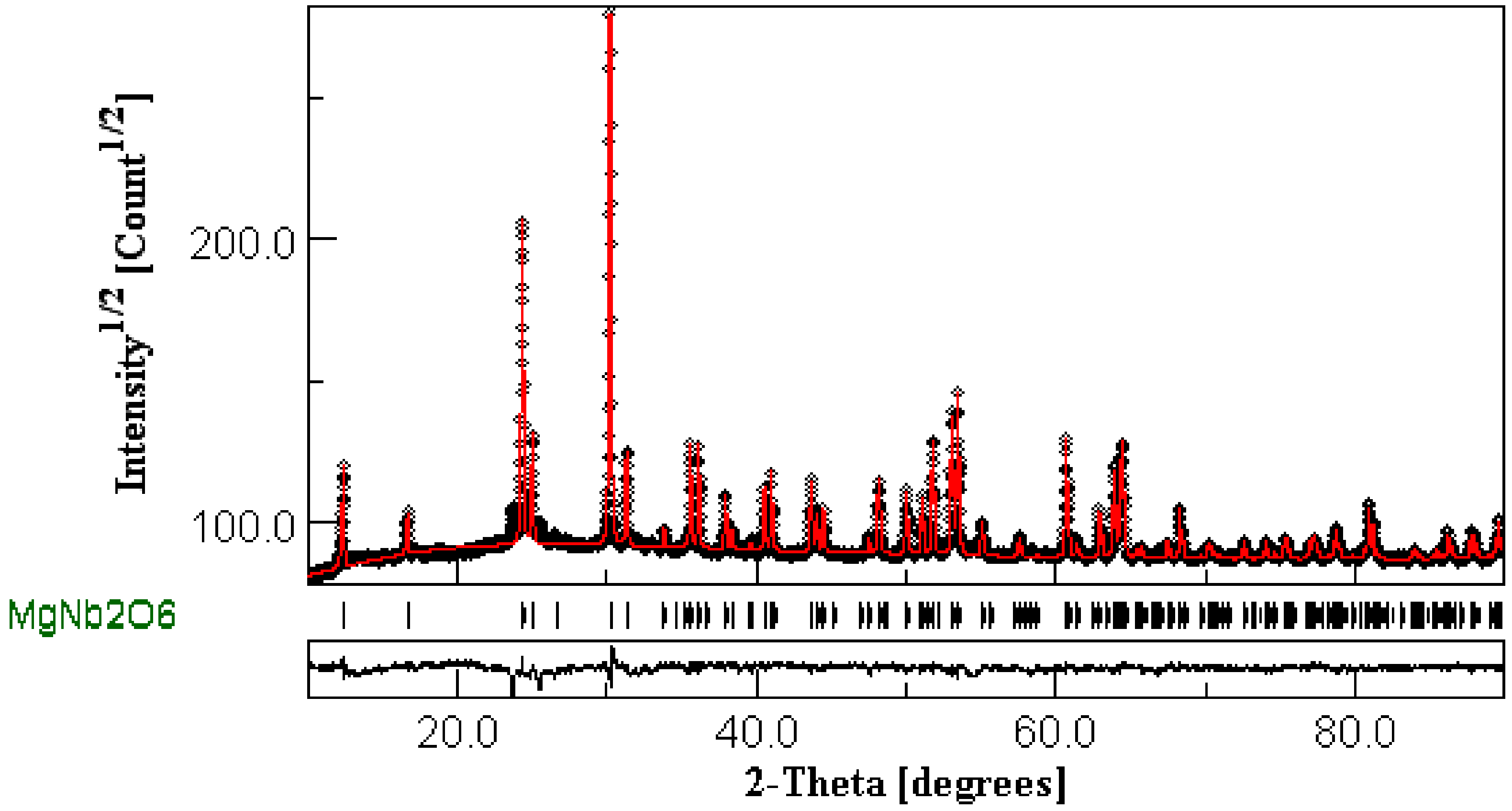

Figure S1. Rietveld refinement of an Ex-situ XRD pattern of a typical MgNb2O6 ternary compound synthesized by solid state reaction of precursors (MgO and Nb2O5) at 1473 K.

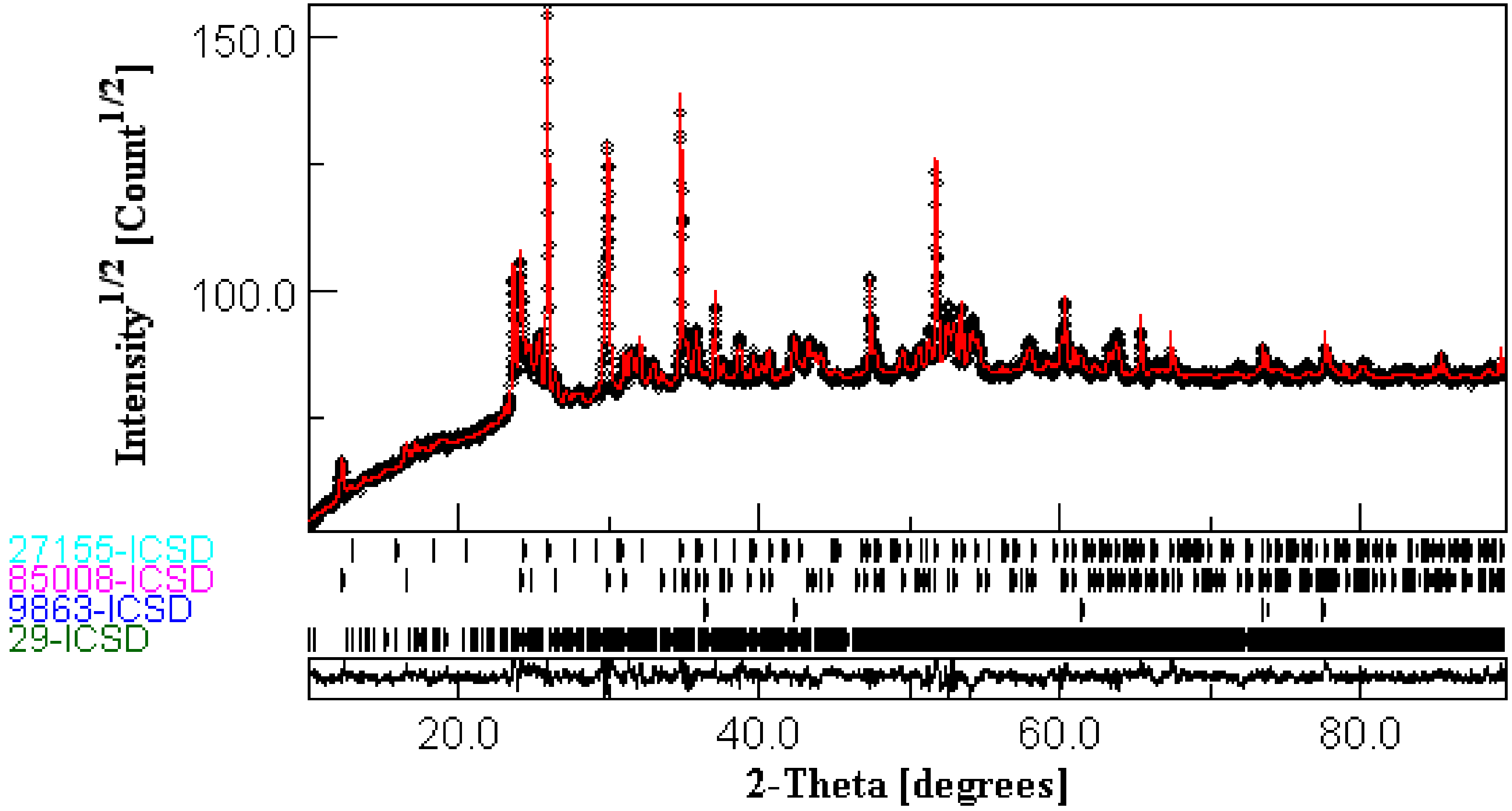

Figure S2. Rietveld refinement of an In-situ XRD pattern of a typical MgNb2O6 ternary compound synthesized by solid state reaction of precursors (MgO and Nb2O5) at 1173 K.
